# Radical Cystectomy and Lymphadenectomy to Two Patients with Pelvic Kidney: Surgical Pitfalls and Considerations

**DOI:** 10.1155/2013/841806

**Published:** 2013-11-04

**Authors:** I. Adamakis, C. Pournaras, I. Katafigiotis, G. Kousournas, E. Fragkiadis, I. Leotsakos, C. Alamanis, D. Mitropoulos, C. Constantinides

**Affiliations:** 1st Department of Urology, Laiko Hospital, University of Athens Medical School, 17 Agiou Thoma Street, 11527 Athens, Greece

## Abstract

Our goal is to describe our experience in the difficulties encountered during radical cystectomy for muscle invasive bladder cancer in patients with contemporary pelvic kidney. Two patients with muscle invasive bladder cancer and contemporary pelvic kidney were subjected to radical cystectomy and extended lymphadenectomy with conversion to an ileal pouch. In both cases, lymphadenectomy was the first step after entering the true pelvis. In order to proceed to the cystoprostatectomy, careful dissection of the ectopic renal vessels and proper mobilization of the kidney were performed. In both cases, an ileal pouch was our choice. The pelvic kidney is the most common sight of renal ectopia. The etiology is the aborted ascent of the fetal kidney from its initial position in the pelvis. This is the first case series describing radical cystectomy for muscle invasive urothelial carcinoma of the bladder in patients with a pelvic kidney.

## 1. Introduction

Renal ectopia is a rare congenital defect which was first described by anatomists in the 16th century. Deriving from the word “ek-topos” which in Greek means “out (of)-place”, and differentiating from the ptotic kidney, the ectopic kidney has never reached its normal position in the renal fossa. The absence or the incomplete cephalad migration and rotation of the metanephric tissue and the ureteral bud in the 8th week of gestation may explain all possible positions of a pelvic, iliac, abdominal, thoracic, contralateral, or crossed ectopic kidney. The most common type of renal ectopia is the pelvic kidney whose incidence varies from 1/500 to 1/1200 among autopsy series. The incidence of pelvic ectopia specifically is estimated in 1/2100 to 1/3000 of autopsies [1].

Renal ectopia is asymptomatic in most patients, explaining its higher incidence in autopsy series compared to the cases with clinical presentation (1/10000) [2]. A prevalence of VUR and pelvic dilatation is estimated at around 20% to 30% with the length of the ureter usually conforming to the position of the kidney. The prevalence of left renal ectopia is slightly higher. The incidence of other congenital anomalies associating the ectopic kidney is rather high with genital anomalies being the most common (15–45%) [3].

The bladder carcinoma is the most common malignancy of the urinary tract, the 9th most common cancer diagnosis worldwide, and its age-standardized incidence rate is 10.1 per 100,000 for males and 2.5 per 100,000 for females [4]. We describe two patients undergoing surgery for invasive urothelial carcinoma in whom an ectopic pelvic kidney was discovered.

## 2. Materials and Methods

A written consent was obtained from both patients.

### 2.1. Case Series

#### 2.1.1. Case  1

**Figure 1:**
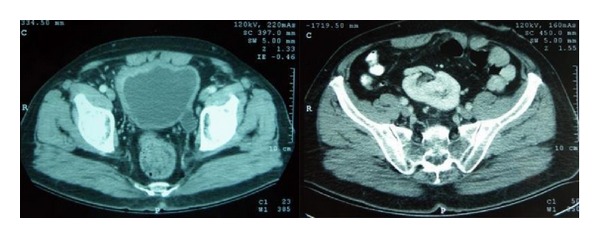
Preoperative CT of case  1: bladder carcinoma preoperative and left pelvic kidney.

**Figure 2:**
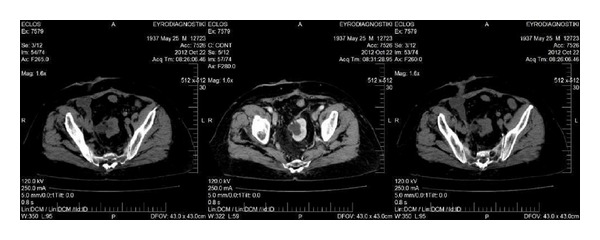
Postoperative CT of case  1.

A 68-year-old man presented with macroscopic hematuria. The diagnostic ultrasound revealed a bladder tumor. The Computed Tomography (CT)-scan showed no prevalent metastatic lesions, while an ectopic left pelvic kidney was discovered (Figure 1). The biopsy showed a high grade muscle invasive urothelial carcinoma. Radical cystoprostatectomy with extended lymphadenectomy was performed with conversion to an ileal pouch (Figure 2). Twenty-one lymph nodes were resected. The final pathology showed a pT2aG3 urothelial carcinoma and multiple sites of urothelial in situ carcinoma. There are no signs of metastatic disease 1.5 years after cystectomy.

#### 2.1.2. Case  2

**Figure 3:**
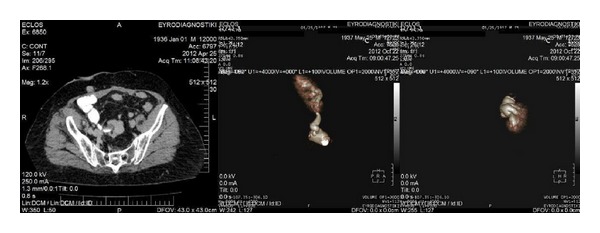
Postoperative and CT-pyelography (3D) of case 2.

A 73-year-old man presented with macroscopic hematuria. A pT1 g3 urothelial carcinoma was diagnosed at initial histology, while a re-TUR Bladder confirmed it. Preoperatively, an ectopic pelvic left kidney was reported. A BCG regimen was started and after 2 years a muscle-invasive bladder carcinoma was diagnosed. Staging CT showed no metastatic lesions. Radical cystoprostatectomy with standard lymphadenectomy, removing all nodal tissue cranially up to the common iliac bifurcation, was performed. Seventeen lymph nodes were excised. The ureters were anastomosed to an ileal conduit (Figure 3). Final pathologic report revealed a pT2 g3 urothelial carcinoma with no involvement of any lymph nodes, while all surgical margins were clear. After 2.5 years of followup the patient remains free of disease.

## 3. Results

In both cases, a left pelvic kidney was discovered during surgical staging, which is in accordance with the highest prevalence of pelvic kidney in the left side. The altered vasculature and the short ureteral length are due to the technical difficulties during radical surgery for urothelial carcinoma of the bladder.

In both cases, lymphadenectomy was the first step after entering the true pelvis. Following the common iliac vessels along to the bifurcation of the aorta in case 1 and clearing the lymphatic tissue around the vessels, a single left renal vein appeared ending up to the proximal inferior vena cava (IVC). A single tortuous left renal artery was revealed, rising from the distal aorta, ending up to the hilus of the pelvic kidney. In order to proceed to the cystoprostatectomy, careful dissection of the ectopic renal vessels and proper mobilization of the kidney were performed. Although the ectopic ureter was relatively short, its length allowed its anastomosis to an ileal conduit on the right side without tension without passing below the mesosigmoid and without disruption of its vascularization. A refluxing end-to-end ureteroileal anastomosis was chosen. Both ureters were spatulated for 0.5–1 cm and the anastomoses were stented with silicon catheters.

In case  2, also starting with removing lymphatic tissue around the iliac vessels, the ectopic vessels of the left kidney were exposed. The left ureter was revealed after recognizing the renal vessels. Blood was supply derived in this case from the left common iliac artery while the left renal vein ended up to the distal IVC. The ectopic left kidney was retracted superiorly and the cystoprostatectomy was completed. The ureters both the ectopic, with its short length, as well as the right one were anastomosed directly to a conduit of distal ileum without further mobilization and were stented again with silicon catheter.

## 4. Discussion

When invasive bladder carcinoma is encountered, an ectopic pelvic kidney is a real challenge for every surgeon irrespective of his experience and technical skills. The specific features of a pelvic kidney including the different length of the ureter and the variable vascularization as well as the changes in the anatomy in the true pelvis are the basic difficulties during the operation for muscle invasive bladder cancer. In both cases, an ileal pouch was our choice. Cutaneous ureterostomy was not an option since the length of the ectopic ureter was short and both patients were overweight. In theory, an orhtotopic ileum-neobladder would be another option, however, no such cases have been reported yet.

Discussing preoperative evaluation of the vasculature in cases of renal ectopia and urothelial or gynecologic malignancies of the pelvis, the options of three-dimensional CT reconstruction or preoperative angiography have been proposed [5]. Although better imaging may prove helpful before surgery, offering information about the kidney's vasculature, rotation, and ureteral length, the choice of the urinary diversion has to be discussed with the patient preoperatively and may depend on the findings during the operation (e.g., length of ureters, length of mesentery).

Three cases of radical cystectomy in the presence of an ectopic kidney have been reported, two of them in a fused pelvic kidney [6–8]. As mentioned before, great care is necessary to preserve the kidney's vasculature during lymphadenectomy and its mobilization to perform cystectomy. An ileal conduit is probably the most safe option in order to have a tension-free anastomosis of the ureters in the presence of a pelvic kidney. In case of a single fused kidney, the length of the ureters is even shorter. Concerning the urinary diversion, in our cases the short ureters of both ectopic kidneys, the patients characteristics (BMI > 30), as well as the presence of a short mesentery (especially in case  1) was the reason we chose the ileal conduit. A neobladder could actually be technically feasible, although the ectopic kidney occupies almost the entire true pelvis. Questions rise whether in case of an orhtotopic neobladder the ureteroileal anastomoses as well as the one of the neobladder to the urethra would be free of tension. The presence of the pelvic kidney could possibly affect the vascularization of the neobladder as well due to the pressure of the pelvic kidney. The specific features of this anatomical variation and the uncertain results of neobladder have been explained to our patients and they chose to have a conversion to an ileal conduit.

## 5. Conclusions

The pelvic kidney is the most common sight of renal ectopia. The etiology is the aborted ascent of the fetal kidney from its initial position in the pelvis. The specific features of a pelvic kidney including the different length of the ureter the variable vascularization as well as the changes in the anatomy in the true pelvis are the basic difficulties occurring during radical cystectomy for muscle invasive bladder cancer. This is the first case series describing radical cystectomy for muscle invasive urothelial carcinoma of the bladder in two patients with a pelvic kidney.
